# Supplementary material for: Data linkage of two national databases: Lessons learned from linking the Dutch Arthroplasty Register with the Dutch Foundation for Pharmaceutical Statistics
Source: PLoS One. 2023 Mar 8;18(3):e0282519. doi: 10.1371/journal.pone.0282519 (PMC9994672; doi:10.1371/journal.pone.0282519)
Supplement: S1 Table — (DOCX) [file pone.0282519.s001.docx]

**Supplementary table 1a** The selected primary arthroplasty population linked on low-molecular-weight heparin and patient postcode compared to the not-linked population

|  | **Not-linked arthroplasties** | |  | **Linked arthroplasty population** | |
| --- | --- | --- | --- | --- | --- |
|  | n = 214 663 | |  | n = 153 808 | |
| **Joint = knee (%)** | 91 546 | 42.6% |  | 73 362 | 47.7% |
| **Sex = Female (%)** | 140 881 | 65.6% |  | 97 629 | 63.5% |
| **Age (mean(SD))** | 70.8 | 10.5 |  | 67.6 | 10.4 |
| **BMI^*^ (%)** |  |  |  |  |  |
| <=18.5 | 2 225 | 1.2% |  | 1 038 | 0.7% |
| 18.5-25 | 52 716 | 29.0% |  | 37 277 | 25.7% |
| 25-30 | 73 516 | 40.4% |  | 60 174 | 41.4% |
| 30-40 | 49 543 | 27.3% |  | 43 390 | 29.9% |
| >40 | 3 798 | 2.5% |  | 3 315 | 2.3% |
| missing | 32 865 | 15.3% |  | 8 614 | 5.6% |
| **Smokers^*^ = Yes (%)** | 58 153 | 10.0% |  | 30 064 | 11% |
| missing | 40 843 | 19.0% |  | 14 768 | 9.6% |
| **Osteoarthritis = Yes (%)** | 173 906 | 82.0% |  | 135 539 | 88.5% |
| missing | 2 548 | 1.2% |  | 653 | 0.4% |
| **Charnley Classification^*^ (%)** |  |  |  |  |  |
| A | 72 501 | 41.9% |  | 60 683 | 44.8% |
| B1 | 48 336 | 27.9% |  | 39 639 | 29.3% |
| B2 | 32 231 | 18.6% |  | 26 154 | 19.3% |
| C | 5 136 | 3.0% |  | 3 282 | 2.4% |
| Not applicable | 14 841 | 8.6% |  | 5 693 | 4.2% |
| missing | 41 618 | 19.4% |  | 18 357 | 11.9% |
| **ASA Classification (%)** |  |  |  |  |  |
| ASA-I | 29 731 | 14.0% |  | 27 281 | 17.8% |
| ASA II | 133 478 | 62.8% |  | 100 386 | 65.5% |
| ASA III-IV | 49 491 | 23.3% |  | 25 685 | 16.8% |
| missing | 1 963 | 0.9% |  | 519 | 0.3% |
| n = number of arthroplasties, SD = standard deviation, BMI = Body Mass Index (kg/m2), ASA= American Society of Anaesthesiologists Physical Status, SMD = Standardized Mean Difference, Charnley A= One joint affected with osteoarthrosis; B1= two joints affected (both hips/both knees); B2= Contralateral joint with prothesis; C= Multiple joints affected with osteoarthrosis or a chronic disease impairing quality of life (in walking). **^*^=** available since 2014 | | | | | |
