# Supplementary material for: Data linkage of two national databases: Lessons learned from linking the Dutch Arthroplasty Register with the Dutch Foundation for Pharmaceutical Statistics
Source: PLoS One. 2023 Mar 8;18(3):e0282519. doi: 10.1371/journal.pone.0282519 (PMC9994672; doi:10.1371/journal.pone.0282519)
Supplement: S2 Table — (DOCX) [file pone.0282519.s002.docx]

**Supplementary table 1b.** The selected revision arthroplasty population linked on low-molecular weight heparin and patient postcode compared to the not-linked population

|  | **Not-linked arthroplasties** | |  |  | **Linked arthroplasty population** | |
| --- | --- | --- | --- | --- | --- | --- |
|  | n = 19 123 | |  |  | n = 12 549 | |
| **Joint = knee (%)** | 7 187 | 37.6% |  |  | 6 004 | 47.8% |
| **Sex = Female (%)** | 12 273 | 64.2% |  |  | 8 032 | 64.0% |
| **Age (mean(SD))** | 71.2 | 10.9 |  |  | 67.7 | 10.7 |
| **BMI^*^ (%)** |  |  |  |  |  |  |
| <=18.5 | 208 | 1.3% |  |  | 102 | 0.9% |
| 18.5-25 | 4 594 | 28.9% |  |  | 2 906 | 24.9% |
| 25-30 | 6 193 | 38.9% |  |  | 4 762 | 40.8% |
| 30-40 | 4 494 | 28.2% |  |  | 3 620 | 31.0% |
| >40 | 431 | 2.7% |  |  | 277 | 2.4% |
| missing | 3 203 | 16.7% |  |  | 882 | 7.0% |
| **Smokers^*^ = Yes (%)** | 5 202 | 9.5% |  |  | 2 686 | 12.2% |
| missing | 3 748 | 19.6% |  |  | 1 317 | 10.5% |
| **Charnley Classification^*^ (%)** |  |  |  |  |  |  |
| A | 6775 | 45.9% |  |  | 5 208 | 49.4% |
| B1 | 2150 | 14.6% |  |  | 1 646 | 15.6% |
| B2 | 3709 | 25.1% |  |  | 2 641 | 25.1% |
| C | 989 | 6.7% |  |  | 523 | 5.0% |
| Not applicable | 1131 | 7.7% |  |  | 518 | 4.9% |
| missing | 4 369 | 22.8% |  |  | 2 013 | 16.0% |
| **Type of revision (%)** |  |  |  |  |  |  |
| Total revision | 5 900 | 31.2% |  |  | 4 307 | 34.5% |
| Partial revision | 12 456 | 65.9% |  |  | 8 018 | 64.1% |
| Other | 540 | 2.9% |  |  | 177 | 1.4% |
| missing | 227 | 1.2% |  |  | 47 | 0.4% |
| **ASA Classification (%)** |  |  |  |  |  |  |
| ASA-I | 1 697 | 9.1% |  |  | 1 638 | 13.3% |
| ASA II | 10 736 | 57.9% |  |  | 8 146 | 66.2% |
| ASA III-IV | 6 119 | 33.0% |  |  | 2 513 | 20.4% |
| missing | 571 | 3.0% |  |  | 252 | 2.0% |
| n = number of arthroplasties, SD = standard deviation, BMI = Body Mass Index (kg/m2), ASA= American Society of Anaesthesiologists Physical Status, Charnley A= One joint affected with osteoarthrosis; B1= two joints affected (both hips/both knees); B2= Contralateral joint with prothesis; C= Multiple joints affected with osteoarthrosis or a chronic disease impairing quality of life (in walking). **^*^=** available since 2014 | | | | | | |
