# Supplementary material for: Data linkage of two national databases: Lessons learned from linking the Dutch Arthroplasty Register with the Dutch Foundation for Pharmaceutical Statistics
Source: PLoS One. 2023 Mar 8;18(3):e0282519. doi: 10.1371/journal.pone.0282519 (PMC9994672; doi:10.1371/journal.pone.0282519)
Supplement: Attachment — Submitted filename: Response to reviewers.docx [file pone.0282519.s003.docx]

Dear Shao-Hsien Liu,

Thank you for the opportunity to revise our manuscript. We want to thank you and the reviewer for taking the time to read and provide feedback on our manuscript which improved the quality. In this letter we explain the adjustments we made in response to these comments. Revisions are also highlighted using ‘track changes’ in the manuscript. Page and line numbers refer to the manuscript

We hope the manuscript is suitable for publication in its current form and look forward to hearing your decision.

Kind regards,

Heather van Brug
(for the authors)

1. Please ensure that your manuscript meets PLOS ONE's style requirements, including those for file naming. The PLOS ONE style templates can be found at

<https://journals.plos.org/plosone/s/file?id=wjVg/PLOSOne_formatting_sample_main_body.pdf> and

<https://journals.plos.org/plosone/s/file?id=ba62/PLOSOne_formatting_sample_title_authors_affiliations.pdf>".

*We have adopted these changes throughout the whole manuscript.*

2. In the ethics statement in the Methods and online submission information, please ensure that you have specified what type you obtained (for instance, written or verbal, and if verbal, how it was documented and witnessed). If your study included minors, state whether you obtained consent from parents or guardians. If the need for consent was waived by the ethics committee, please include this information.

*We have adopted these changes (lines 291-295).*

3. In your Data Availability statement, you have not specified where the minimal data set underlying the results described in your manuscript can be found. PLOS defines a study's minimal data set as the underlying data used to reach the conclusions drawn in the manuscript and any additional data required to replicate the reported study findings in their entirety. All PLOS journals require that the minimal data set be made fully available. For more information about our data policy, please see <http://journals.plos.org/plosone/s/data-availability>.

Upon re-submitting your revised manuscript, please upload your study’s minimal underlying data set as either Supporting Information files or to a stable, public repository and include the relevant URLs, DOIs, or accession numbers within your revised cover letter. For a list of acceptable repositories, please see [http://journals.plos.org/plosone/s/data-availability#loc-recommended-repositories](http://journals.plos.org/plosone/s/data-availability#loc-recommended-repositories). Any potentially identifying patient information must be fully anonymized.

Important: If there are ethical or legal restrictions to sharing your data publicly, please explain these restrictions in detail. Please see our guidelines for more information on what we consider unacceptable restrictions to publicly sharing data: <http://journals.plos.org/plosone/s/data-availability#loc-unacceptable-data-access-restrictions>. Note that it is not acceptable for the authors to be the sole named individuals responsible for ensuring data access.

We will update your Data Availability statement to reflect the information you provide in your cover letter.

“There are legal restriction to sharing our data publicly. In the Netherlands we have a General Data Protection Regulation (wet AVG, GDPR) which is aimed at protecting the data of EU-citizens. The data used, especially the identifiers to link the datasets, contain potentially identifying or sensitive patient information which according the GPDR cannot be shared. Moreover, the data are owned by two independent third-party organizations (Dutch Foundation for Pharmaceutical Statistics (SFK) and the Dutch Arthroplasty Register (LROI)) and the SFK was obliged to destroy the data they received from the LROI. To gain access to the pseudonymized data both the Foundation for Pharmaceutical Statistics and the Dutch Arthroplasty Register would need to be contacted to recreate the linkage. Contact information for both instances: lroi@orthopeden.org (Dutch Arthroplasty Register); [info@sfk.nl](mailto:info@sfk.nl) (Foundation for Pharmaceutical Statistics).

The Statistics Netherlands also has to adhere to GDPR, and is a third-party organization. Data cannot be freely shared with third parties as Statistics Netherlands does not permit this. Data can be accessed by contacting Statistics Netherlands: [microdata@cbs.nl](mailto:microdata@cbs.nl)

4. We note that you have indicated that data from this study are available upon request. PLOS only allows data to be available upon request if there are legal or ethical restrictions on sharing data publicly. For more information on unacceptable data access restrictions, please see [http://journals.plos.org/plosone/s/data-availability#loc-unacceptable-data-access-restrictions](http://journals.plos.org/plosone/s/data-availability#loc-unacceptable-data-access-restrictions).

In your revised cover letter, please address the following prompts:

a) If there are ethical or legal restrictions on sharing a de-identified data set, please explain them in detail (e.g., data contain potentially sensitive information, data are owned by a third-party organization, etc.) and who has imposed them (e.g., an ethics committee). Please also provide contact information for a data access committee, ethics committee, or other institutional body to which data requests may be sent.

b) If there are no restrictions, please upload the minimal anonymized data set necessary to replicate your study findings as either Supporting Information files or to a stable, public repository and provide us with the relevant URLs, DOIs, or accession numbers. For a list of acceptable repositories, please see [http://journals.plos.org/plosone/s/data-availability#loc-recommended-repositories](http://journals.plos.org/plosone/s/data-availability#loc-recommended-repositories).

We will update your Data Availability statement on your behalf to reflect the information you provide.

*We have provided additional information in the cover letter and have answered this point under point 3 in this rebuttal*

5. Please include your full ethics statement in the ‘Methods’ section of your manuscript file. In your statement, please include the full name of the IRB or ethics committee who approved or waived your study, as well as whether or not you obtained informed written or verbal consent. If consent was waived for your study, please include this information in your statement as well.

*We’ve added this to the manuscript (lines 292-295):*

*“****Ethics***

*This study was exempted by the Medical Research Ethics Committee Leiden - Den Haag - Delft from review under the Medical Research Involving Human Subjects Act as it did not apply to the current study. Reference number: G19.018. The need for informed consent was waived.”*

*”*

Abstract:
• Line 27, “To provide guidance on data linkage in case of non-unique identifiers we present….” – suggest adding a coma before we

*Has been adopted*

• Line 38-39, “Representativeness was assessed by comparing the patient postcode-LMWH-group with the other arthroplasties” – what are other arthroplasties that were referring to? Please be specific.

Has been changed to “differently linked” arthroplasties.

• Line 39-40, it is not clear what the external validation meant? Was it referring to the representativeness of the overall sample from the data linkage compared to other sample (generalizability) or any other outputs that authors compared with?

*This is referring to comparing it to one of the major outcomes of our original project, the opioid prescription rates, in the CBS. (lines 39-42).*

• Line 41, we typically do not use numbers to start with a sentence.

*This has been changed. See line 43.*

• Instead of listing your findings, conclusion should present your main results in light of the evidence in your research. Please consider revising the conclusion to reflect main findings as well as the specific strengths and limitations of your research.

*Has been changed, see lines 56-59:
“After identifier selection, checking data availability and internal validity, assessing representativeness, and externally validating our results we found sufficient linkage quality in the patient-postcode-LMWH-group, which consisted of around 40% of the arthroplasties performed after 2013.”*

Introduction:
• It would benefit the readers if authors can provide additional context on what are the common approaches for non-unique identifiers to link databases and how they typically perform. So that later when author present your findings, readers can follow through with better ideas about those approaches.

*The introduction has been updated to further elaborate the non-unique data-linkage. Please see lines 71-73. With regard to the typical performance of non-unique identifier approaches we feel that it is very difficult to provide such information as this is highly dependent on the quality of the data and the availability of discriminative identifiers. Therefore we did not add this to the manuscript.*

• It would be easier for readers to follow if authors can separate the long paragraphs into different sections.

*This has been adopted.*

Methods:
• It is not clear what the external validation meant here in the present manuscript. In general, external validation means to evaluate the extent to which the findings of a study can be applicable to other settings.

*By external validation we mean that we compared the opioid prescription rates we found in the LROI-SFK database with the opioid prescription rates we found in the CBS (another setting). In essence the CBS has similar data available, all arthroplasties performed in the Netherlands with exception of the arthroplasties performed in private hospitals as well as medication data, but less detailed. However linkage in the CBS is performed on social security number. As such the linkage in the CBS should ensure a near-perfect linkage. As we found similar opioid prescription rates we assume sufficient external validity.*

Despite that the current metrics used to compared findings for the main purpose/study questions of the data merging were relevant, the representees and/or characteristics of the merged sample compared to other studies were not provided. In addition, generalizability requires internal validity as well as a judgement on whether the findings of a study are applicable to a particular group.

However, such discussion in the current manuscript seems lacking. A section to describe the process of evaluating internal validation may be helpful.

*Thank you for this comment. We fully agree that generalizability requires internal validity. We used the LROI registry, which provides an over 98% complete database of all arthroplasty surgeries performed in the Netherlands. Hence our main database (LROI database), consisted of the source population as it is the entire population to which the results of our main project are applicable. We have added a supplementary table in which the comparison of our linked population is compared to the other arthroplasties performed since 2013 which were considered not linked (or linked less well). As shown in the manuscript, we believe our sample to be representative for the other arthroplasties, but do see some small differences in: percentage of knee arthroplasties, age, male-female distribution, percentage osteoarthritis patients and the distribution of Charnley and ASA-score, indicating possibly a slightly more healthy population. We’ve also added a reference to a paper of our main project (this was not published yet when we submitted our current manuscript), in which we elaborate on the used population and the representativeness in relation to the source population (Lines 378-381).*

• I would also suggest a separate section for the description of validation process that includes the discussion of potential metrics and/or sample characteristics to evaluate the representativeness of sample from the merged database.

*This has been adopted. We added information to the paragraph on assessing representativeness. (Lines 266-273.)*

• The rationale behind probabilistic versus deterministic linkage was not clear. For example, probabilistic linkage could be a more accurate method in poorer quality data. However, such context or information was not provided in the paper.

*Thank you for this comment. We’ve added this to the manuscript (lines: 116-127).*

• The information regarding the specific discriminative power (from basic set to additional variables added) were not available in the paper. To our knowledge, the performance of deterministic linkage could be impacted by reduced discriminative power. Information on how much the discriminative power were improved could be beneficial to the readers.

*The manuscript included some information on the discriminative ability that we were able to elaborate on in relation to the linkage based on the discriminative ability of for example postcode area (lines 184-188). We’ve further elaborated with information on the number on non-unique links* (see lines: 189-193; 300-307). Unfortunately *we cannot provide any additional information as the linkage has been performed by the SFK and per our agreement (based on legal obligations) the data have been deleted after the linkage was performed.*

• The rationale behind the of use thromboprophylaxis as a potential identifier was not clear.
*As all patients receive thromboprophylaxis after arthroplasty surgery, we used these prescriptions* *to add discriminative identifiers to these patients. As the SFK holds all Dutch patients additional information (birthyear, sex, and 4-digit postcode) this was needed because without the thromboprophylaxis it was not possible to create unique links. We’ve also added an international reference to underline this choice.*

• Line 160-168, this section reads more like background information on why authors used those measures to assess quality of data linkage rather than the description of approaches. Those may be more appropriate in the intro or discussion sections.

*Thank you for this comment, we have taken the liberty to leave it like this. We feel this text introduces the thoughts we had regarding the checking of the data quality and is a good introduction of the three ; steps we used to assess the linkage quality. We did added some extra explanation in* the introduction lines 84-86:

“. In the methods section the different steps to link these databases will be discussed, each section introduced with a short explanation of the steps.”

Results:
• Based on the findings from initial linkage rate which was about 43-48%, I wonder authors have information on what main identifiers were missing from the sample that those data could not be linked?

*It is difficult to assess which factors were missing, as we do not have this information (this has been added to the discussion (lines 445-456)). However, we know that if patients would not have collected their thromboprophylaxis within the dedicated timeframe they would not have been eligible for linkage (and therefore would not be linked) for instance, patients with a prolonged hospital stay, or patients discharged to nursing homes. Additionally, there was a proportion of arthroplasties not linked to multiple arthroplasties (now added on lines 300-307). This could have been related to additional factors, perhaps many elderly living in the same area.*

• Line 289-291, the results corresponding to the description here were not provided anywhere in the manuscript.

*This has been added with a reference to where these results are published.*

• Line 293-298, same comments above. Either a figure and/or numbers representing that information was missing.

*This has been added with a reference to where these results are published.*

Discussion:
• The first paragraph of discussion (line 308-326) mainly focused on the 10% linkage uncertainty from group 3. However, it would be helpful that authors add appropriate references/citations to support your explanations or arguments here in the section.

*Thank you for this comment, we’ve added additional references. However, one of these is in Dutch. An English translation, provided by us, the authors, of the specific text is inserted below:*

*“To combine the data from persons in different pharmacies, the SFK initiated a collaboration with ZorgTTP. This is a Trusted Third Party, also known as a digital notary. ZorgTTP offers, within legal limits, the possibility to create an individual pseudonym. The SFK receives theses pseudonyms from ZorgTTP which allows them to follow patients, anonymously, over multiple pharmacies. Using these pseudonyms, the SFK is able to, more accurately, count individual patients.”*

• Line 336-351, is there any data or published information to support the findings from CBS data regarding opioid prescriptions. Currently, no data was presented or provided with a reference to support those discussions.

*We’ve added the reference to support these findings, we hope this provides enough information to support the claims.*

Tables & Figures:
• Some components belong to the evaluation of internal validation, and some are for external validation. It would be helpful to provide subheading with corresponding sections on those steps regarding the data linkage process.

*We’ve adjusted table 1 to reflect the difference in the internal-/external validation process. We’ve further added subheadings to aid the reader.*

• Table 3: instead of group 1, 2 ,3, it would be beneficial to the readers if authors can list/name those groups directly in the column.
*This has been adopted.*
