# Supplementary material for: Data linkage of two national databases: Lessons learned from linking the Dutch Arthroplasty Register with the Dutch Foundation for Pharmaceutical Statistics
Source: PLoS One. 2023 Mar 8;18(3):e0282519. doi: 10.1371/journal.pone.0282519 (PMC9994672; doi:10.1371/journal.pone.0282519)
Supplement: Attachment — Submitted filename: Response to reviewers.docx [file pone.0282519.s004.docx]

Dear Dimitrios Sokratis Komaris,

Thank you for the opportunity to revise our manuscript. We want to thank you and the reviewers for taking the time to read and provide feedback on our manuscript which improved the quality. In this letter, we explain the adjustments we made in response to these comments. Revisions are also highlighted using ‘track changes’ in the manuscript. Page and line numbers refer to the manuscript

We hope the manuscript is suitable for publication in its current form and look forward to hearing your decision.

Kind regards,

Heather van Brug
(for the authors)

Review Comments to the Author

Dear reviewer,

Thank you for reviewing our manuscript and for providing constructive feedback which improved our manuscript.

Reviewer #1: In my initial review I expressed concern about the assumption that all TKA and THA patients get chemoprophylaxis for VTE. I indicated that this claim should be supported because elsewhere in the world there has been a large shift to aspirin, which would not show up in SFK data. The authors seem to have responded by adding Reference 13 on line 133. However, reference 13 appears to be "An analysis of the relocation behavior of people in their sixties" that is not relevant to the VTE prophylaxis issue. The reference should be corrected.

*This reference was indeed incorrect, thank you for the attention to detail. We have updated the reference to the correct page. This reference is now the pharmacotherapeutic compass (a Dutch website) which guides doctors in the prescription of medication.*

Reviewer #2: Dear Editor, Dear Author

Dear Leslie Grammatico-Guillon,

Thank you for reviewing our manuscript and for providing constructive feedback which improved our manuscript.

First the subject of the article is of great interest for the future use of Big data in healthcare surveillance and research.
However, the writing and understanding of the research performed could be synthetized and hence clarified. Indeed, the second sentence has no verb! In the second paragraph the tense of the verbs must also be homogenized.

*Thank you for pointing this out. We’ve corrected our English.*

Moreover, Netherlands is lucky with his unique patient number over the country for most of their healthcare databases. Which is not the case in a lot of European countries.
And the key issue of non-unique identifiers is less important in their reuse as compared with other countries.

*Thank you for the interesting thought. We agree that this issue is even more important in other countries where unique patient numbers are not available in most healthcare databases. In regards to the unique patient number in the Netherlands, it is important to note that in the Netherlands this often is not the case as well.*

The definition of case study, in informatics we would probably call it use case in orthopedics more than case study, in my opinion.

*In regards to your suggestion to change our definition to a “use case” instead of a “case-study”, we took the liberty to leave the term case-study in. Within orthopedics, the term use case is not often adopted. Moreover while traditional case studies in the medical field are often case reports, currently methodological issues are also addressed as case-study in the case of a detailed study of a specific method. For example:*

*Blanchette, C., Dekoven, M., De, A., Roberts, M., 2013. Probabilistic data linkage: a case study of comparative effectiveness in COPD. Drugs in Context 1–5.. doi:10.7573/dic.212258*

*Resnick, D.M., Cox, C.S., Mirel, L.B., 2021. Using synthetic data to replace linkage derived elements: a case study. Health Services and Outcomes Research Methodology 21, 389–406.. doi:10.1007/s10742-021-00241-z*

The article is interesting but must been shortened and synthetize to go straight to the point, with less chapter titles and short paragraphs.

*We have reduced the length by 500 words. We hope that the revised version is more focused and easier to read.*

The discussion must also be shortened, to be suitable to publish as a use case.

*We have reduced the discussion.*
